# Comprehensive analysis of the leukocyte immunoglobulin-like receptor family in clear cell renal cell carcinoma

**DOI:** 10.1080/07853890.2025.2546684

**Published:** 2025-08-22

**Authors:** He Wei, Zixiang Cong, Chengtao Niu, Xintong Sun, Zhongshun Yao, Yiming Zhang, Xue Jiang, Zhihong Niu, Qiang Fu

**Affiliations:** ^a^ Department of Urology, Shandong Provincial Hospital Affiliated to Shandong First Medical University, Jinan, Shandong, China; ^b^ Department of Urology, Weihai Municipal Hospital Affiliated to Shandong University, Weihai, Shandong, China; ^c^ Department of Urology, Shandong Provincial Hospital, Shandong University, Jinan, Shandong, China; ^d^ Department of Emergency, Liaocheng People’s Hospital, Liaocheng, Shandong, China; ^e^ Department of Urology, People’s Hospital of Changle County, Weifang, Shandong, China; ^f^ Medical school, Shandong Xiehe University, Jinan, Shandong, China

**Keywords:** Leukocyte immunoglobulin-like receptor, signature, clear cell renal cell carcinoma, prognosis, tumor immune microenvironment

## Abstract

**Background:**

Renal cell carcinoma (RCC) is a malignant tumor originating from the renal tubular epithelium and is the 14th most common cancer worldwide, causing around 180,000 deaths annually. Clear cell renal cell carcinoma (ccRCC) is the predominant subtype, accounting for 70–80% of cases. The potential of the leukocyte immunoglobulin-like receptor (LILR) family members in tumor progression and immune evasion has garnered significant attention.

**Objective:**

To construct a prognosis signature based on the LILR family in ccRCC patients, and explore its relationship with clinical pathological features, immune microenvironment, and drug sensitivity.

**Methods:**

We analyzed the expression and prognostic value of the LILR family in ccRCC, developed a risk scoring model, and examined its correlation with clinical features, immune infiltration, and treatment efficacy. The study also explored the expression and impact of the core gene LILRB3 on ccRCC.

**Results:**

Our findings indicate that the aberrant expression of LILR family members in ccRCC is associated with tumorigenesis and inhibition of anti-tumor immunity. The risk model enhances long-term survival predictions and supports personalized therapies.

**Conclusions:**

These insights establish a foundation for further research into their potential as targets for cancer immunotherapy, improving predictive capabilities and the development of personalized treatments.

## 1. Introduction

Renal cell carcinomaignancy originating from the epithelium of the renal tubules, ranks as the 14th most common cancer globally, clai (RCC), a malming approximately 180,000 lives annually [1]. Clear cell RCC (ccRCC), the predominant pathological subtype of RCC, accounts for an estimated 70–80% of cases [2]. Currently, partial nephrectomy or radical nephrectomy remains the principal treatment modality for patients with early-stage ccRCC. Due to the insidious nature of its symptoms, about a quarter of ccRCC patients are already in a locally advanced or metastatic stage at initial diagnosis [3]. Thanks to rapid advancements in the development of targeted and immunotherapeutic drugs, the long-term survival rates of patients with metastatic renal cell carcinoma have significantly improved, yet challenges such as drug resistance, side effects, and tumor heterogeneity persist [2]. Hence, identifying and exploring new tumor molecular markers and developing effective prognostic prediction tools are crucial for the treatment of clear cell renal carcinoma.

The Leukocyte Immunoglobulin-Like Receptor (LILR) family, also known as the Immunoglobulin-Like Transcript (ILT) family, is a group of receptors characterized by extracellular immunoglobulin domains, playing an immunoregulatory role in both innate and adaptive immunity [4]. LILRs are type 1 transmembrane glycoproteins, comprising 13 family members divided into two subfamilies based on their associated motifs: the LILRA subfamily, linked with the Immunoreceptor Tyrosine-based Activation Motif (ITAM), and the LILRB subfamily, associated with the Immunoreceptor Tyrosine-based Inhibition Motif (ITIM) [5]. LILRs signal through associated ITAM-bearing tumor-associated macrophages or ITIM, mediating immune activation or inhibition signals, respectively. Primarily expressed on bone marrow antigen-presenting cells (APCs) including dendritic cells (DCs), macrophages, and B cells, the balance between LILRA and LILRB under physiological conditions helps maintain immune homeostasis, preventing hyperactivation or autoimmune diseases [6]. In pathological contexts, substantial research supports their key role in bacterial, viral, and parasitic infections, autoimmune and neurodegenerative diseases, transplantation immunity, and cancer [4–7]. Recent studies have shown that, beyond their presence in most leukocyte subgroups, LILR family members may also be aberrantly expressed on cancer cells to support tumorigenesis and suppress anti-tumor immunity, positioning LILRs as potential targets for cancer immunotherapy [8,9].

We conducted a comprehensive analysis of the expression differences and prognostic value of the LILR family in ccRCC, identifying prognostic features of ccRCC patients based on the LILR family and establishing a risk scoring model on this basis. We also explored the correlations between risk scores, clinical features, immune cell infiltration levels, and the efficacy of chemotherapy and immunotherapy. These findings may enhance our ability to predict the long-term survival of ccRCC patients and facilitate the development of personalized treatment approaches.

## 2. Materials and methods

### 2.1. Data acquisition

By accessing the Genomic Data Commons (GDC) portal, we downloaded transcriptome data and prognostic information related to ccRCC (clear cell renal cell carcinoma). After excluding samples lacking either transcriptome data or prognostic information, this study ultimately selected 530 ccRCC samples from the TCGA-KIRC dataset and 72 adjacent normal tissue samples as the control group. Furthermore, by accessing the GEO database, we obtained gene expression matrices for ccRCC and corresponding adjacent normal tissue samples from four datasets: GSE15641, GSE36895, GSE40435, and GSE53757. The GSE22541 dataset contains survival information for 67 patients with primary ccRCC. The Clinical Proteomic Tumor Analysis Consortium (CPTAC, https://cptac-data-portal.georgetown.edu/) database is a major proteomics research project and database resource initiated by the National Cancer Institute (NCI) of the United States. At the protein level, we obtained protein abundance data for 85 patients’ renal tumors and adjacent normal renal tissues from the CPTAC database [10,11]. The TCGA, GEO, ArrayExpress and CPTAC databases have explicitly stated that their sample and data collection processes were conducted under rigorous ethical oversight, with all participants providing informed consent to allow the use of their data for extensive genomic research and public sharing [11–14]. The utilization of these public databases in this study was approved by the Ethics Committee of Shandong Provincial Hospital (approval number: SWYX: NO.2024-430). The expression levels of all genes were converted to TPM (Transcripts Per Million) format and subjected to binary logarithmic transformation to standardize the data and mitigate the impact of extreme values. We excluded genes with missing expression data and removed genes with TPM values less than 1 in more than 25% of the samples to prevent false-positive results. Following an exhaustive literature review, we identified a list of LILR family genes (Supplementary Table 1) [4].

### 2.2. Analysis of gene correlations and protein interactions within the LILR family

Utilizing the “RCircos” package, we generated Circos 2D track images to visualize the localization information of the LILR family genes on human chromosomes [15]. The “ggplot2” package was employed to construct chord diagrams, visualizing the correlations in gene expression levels among the LILR family genes [16]. The GeneMANIA database (https://genemania.org/) was leveraged to explore the genes most closely related to the functional associations of the LILR family genes, and the top 20 genes with the closest relationships were visualized [17]. Furthermore, using the STRING database (https://string-db.org/) version 11.0, we constructed a protein-protein interaction (PPI) network for the LILR family genes. Proteins with a combined score exceeding 0.4 were selected for visualization [18].

### 2.3. Differential gene expression analysis

We utilized the DESeq2 package to assess the differential expression of genes (DEGs) between tumor and non-tumor tissues [19]. LILR family genes with an absolute log2FC > 1 and an adjusted *p*-value < 0.05 were identified as DEGs. The “ggplot2” package in R was used to create boxplots, while the “pheatmap” package was employed for generating heatmaps. Additionally, the “UpSetR” package was used to visualize differential expression of LILR family genes across multiple datasets.

### 2.4. Construction and validation of a LILR-associated risk scoring model

Employing the ‘caret’ package, the 530 instances retrieved from the TCGA database were systematically partitioned into training and validation cohorts following the principles of randomization [20]. We integrated ten distinct machine learning algorithms to evaluate 101 algorithm combinations [21]. These algorithms include Support Vector Machine (SVM), Least Absolute Shrinkage and Selection Operator (Lasso), Gradient Boosting Machine (GBM), Random Forest, Elastic Net, Stepwise Cox, Ridge, CoxBoost, Super Partial Correlation (SuperPC), and Partial Least Squares with Cox regression (plsRcox). Predictive models were developed within the TCGA training cohort and validated across three datasets: TCGA validation set, E-MTAB-1980, and GSE22541. The concordance index (C-index) was calculated for model selection. The optimal model was used to compute LILR-associated risk scores (LARS) for each patient in the TCGA training set, TCGA testing set, E-MTAB-1980, and GSE22541 datasets, stratifying patients into high-risk and low-risk groups based on median risk scores. The model with the highest average C-index across all cohorts was defined as the optimal model. To verify the model’s prognostic predictive capability, Kaplan-Meier (K-M) survival curves were employed to assess significant differences in long-term survival between high-risk and low-risk groups. Time-dependent receiver operating characteristic (ROC) curves were utilized to evaluate the predictive performance of the risk prediction model [20].

### 2.5. Correlation analysis of clinicopathological characteristics in LARS

Initially, univariate and multivariate Cox regression analyses were conducted to identify independent prognostic factors influencing the outcomes of ccRCC patients. In the univariate Cox regression analysis, factors with a *P*-value less than 0.05 were considered statistically significant and included in the multivariate Cox regression analysis. In the multivariate Cox regression analysis, factors with a *P*-value less than 0.05 were further confirmed as independent prognostic factors. The R package “rms” was utilized to construct a prognostic nomogram, visually representing the relationship between independent prognostic factors and long-term survival rates of patients. Calibration plots were drawn to assess the reliability of the prognostic nomogram by comparing the predicted probabilities with the actual observed probabilities.

### 2.6. Pathway enrichment analysis with LILRs

The “ggrepel” package was employed to construct the volcano plot [22]. GO and KEGG analyses were performed using the “clusterProfiler v4.4.4” package [23]. Genes with |log2FC| > 1 and *p* < 0.05 between different LARS risk groups were selected for GO analysis, and the top eight enriched pathways with the smallest adjusted p-values from the “BP”, “CC”, and “MF” categories were visualized.

### 2.7. Immune infiltration analysis

Single-sample gene set enrichment analysis (ssGSEA) is a method designed to evaluate the enrichment of genomic features within individual samples, thereby providing a comprehensive understanding of the infiltration levels of 23 distinct immune cell types in each sample. The Gene Set Variation Analysis (GSVA) package significantly simplifies the implementation of ssGSEA, facilitating the assessment process [24]. The ESTIMATE algorithm employs specific gene expression signatures to quantify the non-cancerous components within tumor tissues [25]. The immune score reflects the extent of immune cell infiltration, while the stromal score indicates the abundance of stromal cells within the tumor microenvironment. These scores are combined in the ESTIMATE score to estimate tumor purity. Tumor Immune Dysfunction and Exclusion (TIDE) analysis is a computational method used to predict cancer patients’ responses to immune checkpoint blockade therapies. The TIDE analysis is conducted through the “Response Prediction” module available at http://tide.dfci.harvard.edu/, calculating dysfunction scores based on the expression levels of intratumoral biomarkers [26]. The Cancer Immunome Database (TCIA) provides comprehensive immunogenomic analysis results from next-generation sequencing (NGS) data for 20 solid cancers from TCGA and other data sources [27,28]. The Immunophenoscore (IPS) is a scoring system used to assess a patient’s immune phenotype. IPS evaluates the activity of different immune cell types, including antigen-presenting cells, effector cells, inhibitory cells, and checkpoint inhibitory molecules, by calculating the expression levels of immune-related genes, where higher scores are associated with increased immunogenicity [29].

### 2.8. Drug sensitivity analysis

Our drug sensitivity study utilizes the publicly available Connectivity Map database (CMap; https://portals.broadinstitute.org/cmap/) for gene expression analysis post small molecule compound application to human cells. We identified 150 DEGs from the high-risk group, ranked by |log2FC| and were analyzed in Cmap [30]. The Normalized Connectivity Score (NCS) shows the correlation between drug and disease perturbation gene expression. Negative and positive scores indicate inhibitory effects on high-risk and low-risk patients respectively. Considering the crucial role of TKIs in the treatment of ccRCC, we have visualized the entries containing TKIs in the CMap analysis results. The criterion used was |NCS| > 1 and *p* < 0.05. In addition, the Genomics of Drug Sensitivity in Cancer (GDSC; https://www.cancerrxgene.org/) database was selected as the primary reference for tumor drug sensitivity data. The GDSC provides an extensive collection of molecular and pharmacological profiles across a wide array of cancer cell lines, thereby facilitating a comprehensive and systematic assessment of drug responses. Drug sensitivity analyses were conducted utilizing the core algorithms implemented in the oncoPredict and pRRophetic packages [31,32]. Initially, gene expression data from study samples were rigorously processed and aligned with the internal reference datasets supplied by both packages. Leveraging these computational frameworks, the sensitivity of each sample to various tyrosine kinase inhibitors (TKIs) was accurately predicted. Specifically, the sensitivities of 20 distinct TKI compounds catalogued within the GDSC were comparatively analyzed between high-risk and low-risk groups. For each cohort, the predicted drug response scores were subjected to statistical comparison, thereby enabling the identification of candidate therapeutic agents exhibiting significantly differential efficacy. Meanwhile, we also analyzed the study by Braun et al. which included 311 patients from the CheckMate-009, CheckMate-010, and CheckMate-025 trials with available clinical, molecular, and immuno-oncological data. This analysis focused on evaluating the sensitivity to two targeted therapies: nivolumab and everolimus [33].

### 2.9. Identification of hub genes

Random forest, by creating multiple decision trees and integrating their prediction results to make final decisions, was used to screen for core genes. First, gene expression data and survival data from ccRCC patients in the TCGA database were integrated. The “sample” function was used to randomly divide all samples into two cohorts: a training set (70%) and a test set (30%). The random seed was set to 123 to ensure reproducibility of the results and to determine the number of decision trees required to achieve the highest accuracy. The “randomForest” function was used to evaluate the error rates of the training and validation sets. Finally, the importance of each variable was assessed by calculating the mean decrease in Gini index and the mean decrease in accuracy.

### 2.10. Patient samples and cell lines

In this investigation, we employed the HEK-293 cell line derived from human embryonic kidney tissue, alongside two distinct renal carcinoma cell lines, Caki-2 and A498, for experimental analyses. The cell lines were obtained from Procell (Procell Life Science & Technology Co Ltd, Wuhan). Additionally, we procured fresh tumor specimens and adjacent non-neoplastic tissue from ccRCC patients who underwent surgical intervention at Shandong Provincial Hospital. This study was conducted with approval from the Medical Ethics Committee of Shandong Provincial Hospital and in accordance with the Declaration of Helsinki, with written informed consent obtained from each participant (approval number: SWYX: NO.2024-430). The paraffin-embedded tissues were re-embedded into novel tissue blocks for immunohistochemical staining. Pathological specimens and clinicopathological characteristics were collected with complete anonymization. All tissue specimens underwent histological diagnosis by two independent pathologists.

### 2.11. Lentiviral vector construction and cell transfection

In the subsequent experiments, the control sequence (NC) was constructed as follows: 5′-TTCTCCGAACGTGTCACGT-3′; LILRB3 shRNA 1:5′-GTGGAGGTTCACATGCTATTA-3′; LILRB3 shRNA 2:5′-GACACTTTCCTTCTGACCAAA-3′; LILRB3 shRNA 3:5′-GACAGAAATAACCCACTGGAA-3′. The lentiviral vectors were constructed by Genechem Packaging Company.The interference sequences were used to transduce cells with concentrated virus, and stable clones were selected with puromycin (Sigma) for two weeks. As described below, the knockdown of LILRB3 expression at the mRNA level was confirmed by qRT-PCR.

### 2.12. qRT-PCR

Total RNA was extracted from cells using TRNzol Universal reagent (DP424, Tiangen Biotech, Beijing, China). The RNA was reverse transcribed into cDNA and subjected to quantitative PCR (qPCR) on a LightCycler96 PCR system (Roche) utilizing SuperReal PreMix Plus (SYBR Green) (FP205, Tiangen Biotech, Beijing, China). Primer sequences are detailed in Supplementary Table 2. The cycling conditions were as follows: 95 °C for 15 min, followed by 40 cycles at 95 °C for 10 s and 60 °C for 30 s. Each sample was analyzed in triplicate with a reaction volume of 20 μl. β-Actin served as an internal control to normalize gene expression, and relative expression levels were calculated using the 2-ΔΔCT method.

### 2.13. Immunohistochemical (IHC) staining

Pathological samples were selected from three patients who underwent radical nephrectomy at the Affiliated Provincial Hospital of Shandong First Medical University between January 1, 2024, and April 1, 2024. Immunohistochemical analysis was conducted using a standardized immunoperoxidase staining method, with samples observed under magnifications of 200× (10× eyepiece, 20× objective) and 400× (10× eyepiece, 40× objective). The staining results were evaluated and corresponding IHC scores were calculated by two independent observers. Positive determination and grading were based on HSI (Hue, Saturation, Intensity): weak positive light yellow scored as 1, moderate positive brown-yellow scored as 2, and strong positive brown scored as 3. The primary antibody was rabbit monoclonal antibody LILRB3 (Proteintech, USA), and the secondary antibody was enzyme-labeled goat anti-rabbit IgG (Servicebio, Wuhan, China).

### 2.14. Western blotting

First, rinse the cells 2–3 times with PBS, then directly add an appropriate amount of 1× SDS-PAGE protein loading buffer. Thoroughly scrape the cells with a cell scraper, and heat the lysate at 100 °C in a metal bath for 5–10 min to extract proteins. Subsequently, determine the protein concentration using the bicinchoninic acid (BCA) method. Mix the protein samples with 5× SDS-PAGE loading buffer at a ratio of 4:1, ensuring thorough vortexing for homogeneity. Load the mixture onto an SDS-PAGE gel for electrophoretic separation, then transfer it onto a polyvinylidene fluoride (PVDF) membrane. Dilute the primary antibody with Western primary antibody dilution buffer at an appropriate ratio and incubate overnight at 4 °C. During overnight incubation, probe the PVDF membrane with primary antibodies against LILRB3 (18260-1-AP, 1:1000, Proteintech, USA), PD-L1 (66248-1-lg, 1:1000, Proteintech, USA), PI3K (67071-1-lg, 1:1000, Proteintech, USA), p-PI3K (Bs-6417R, 1:1000, bioss), AKT (10176-2-AP, 1:1000, Proteintech, USA), p-AKT (80455-1-RR, 1:1000, Proteintech, USA), mTOR (66888-1-lg, 1:1000, Proteintech, USA), p-mTOR (67778-1-lg, 1:1000, Proteintech, USA), and actin (TA-09, 1:1000, ZSBB-Bio, China). Subsequently, dilute the secondary antibody with Western secondary antibody dilution buffer at a 1:2000 ratio. Incubate at room temperature on a shaker for 1 h, followed by washing with 1× TBST three times, each for 10 min. Finally, place the PVDF membrane in a chemiluminescent imaging system, apply a 1:1 mixture of exposure solution A and B evenly onto the membrane, and proceed with exposure. Refer to Supplementary Figure 1 for gel/blot images.

### 2.15. CCK-8 analysis

Cell proliferation was assessed using the Cell Counting Kit-8 (CCK-8) (Meilunbio, Dalian). Cells were seeded in 96-well plates at a density of 5 × 10³ cells per well and allowed to adhere. Subsequently, 10 μl of CCK-8 solution was added to each well, and the cells were incubated at 37 °C in a 5% CO2 atmosphere for 2 h. The absorbance at 450 nm was measured using a microplate reader to determine cell proliferation. Cell proliferation curves were plotted based on the absorbance values at each time point. Experiments were performed in triplicate.

### 2.16. Colony formation assay

When the cells reach the logarithmic growth phase, digest them with trypsin. After centrifugation, prepare a cell suspension and perform cell counting. Add cells at concentrations of 50, 100, and 200 cells per well to 6 cm dishes, mix thoroughly, and incubate at 37 °C in a 5% CO_2_ incubator. Change the medium every three days and continue culturing for approximately 10 days. Add 1 ml of 0.1% crystal violet staining solution to each well and stain for 30 min. Analyze and calculate the number of colonies using ImageJ software.

### 2.17. Migration assays

After digesting the cells, resuspend them in culture medium to prepare a single-cell suspension. Following cell counting, adjust the cell concentration to 2.5 × 10^5^ cells/ml. Assemble the Transwell migration chambers (8 μm pore size, Corning, NY, USA) in a 24-well plate. Add 500 μl of medium containing 10% FBS to the lower chamber and 300 μl of serum-free medium containing the cells to the upper chamber. Incubate at 37 °C for 24 h, then wash with PBS. Fix with 4% paraformaldehyde solution for 30 min and wash three times with PBS. Stain with 0.1% crystal violet solution for 10 min, rinse with running water, and observe and capture images under a fluorescence microscope. Randomly select three fields of view (100×, 200×) for each specimen, count, and calculate the average.

### 2.18. Statistical approaches

Statistical analysis is executed employing R 4.2.1 software [34]. *In vitro* experiments were performed in triplicate. We employed the Pearson correlation algorithm to investigate the correlation between numerical variables, and utilized the Wilcoxon rank-sum test to test the difference between two independent samples. In this study, a default significance level of adjusted *p*-value less than 0.05 is considered statistically significant, unless otherwise specified.

## 3. Results

### 3.1. Correlation analysis of LILR family members

**Figure 1.**
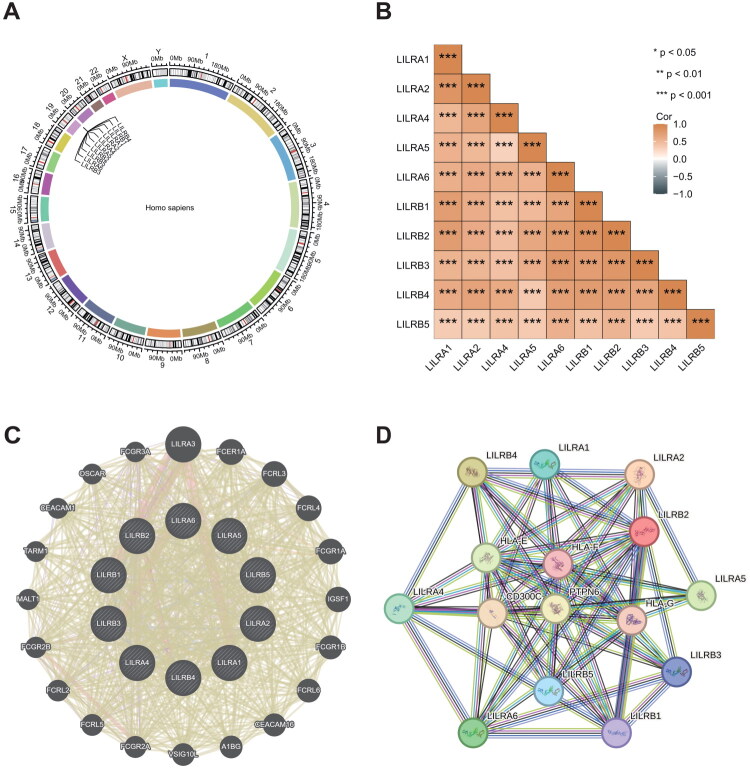
(A) The LILR family genes are clustered within the chromosomal region 19q13.4. (B) Spearman analysis was conducted to assess the correlation among mRNA expression levels of the LILR family genes. (C) GeneMANIA analysis was utilized to explore functional predictions and gene interaction networks of the LILR family genes. (D) PPI analysis was conducted to investigate the protein interactions within the LILR family.

As illustrated in Figure 1A, the genes encoding the LILR family are clustered within the chromosomal region 19q13.4. To further investigate the correlation of gene expression levels within the LILR family, we performed a Spearman correlation analysis and visualized the results. As depicted in Figure 1B, the expression levels of LILR family genes exhibit positive correlations with one another. GeneMANIA was employed to predict the functions of LILR family genes and analyze their gene interaction network, revealing a close association with the FCGR and FCRL families (Figure 1C). Protein-protein interaction (PPI) analysis indicates extensive protein interactions within the LILR family and a significant relationship with the Human Leukocyte Antigen (HLA), suggesting a profound connection between LILR and human immune regulation (Figure 1D).

### 3.2. Identification of differentially expressed genes of the LILRs in ccRCC

**Figure 2.**
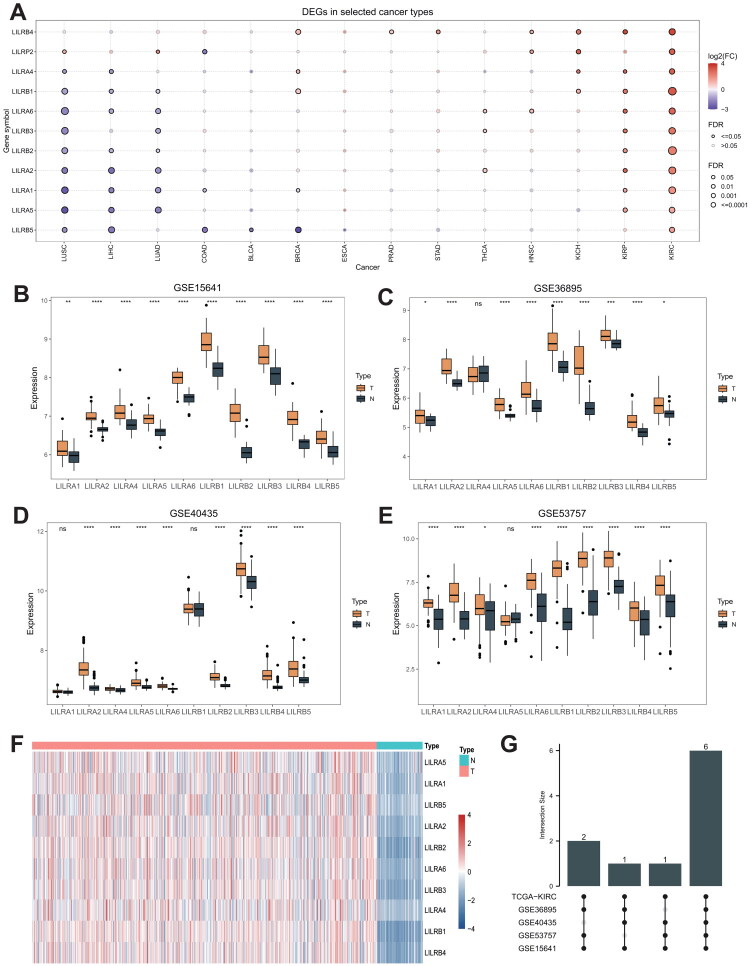
(A) The GSCA database was utilized to explore the differential expression of LILR family genes across various cancers. (B-E) Differential expression of the LILR family in the datasets GSE15641, GSE36895, GSE40435, and GSE53757. (F) a heatmap illustrating the differential expression of the LILRs in the TCGA-KIRC cohort. (G) An upset plot indicating that six genes exhibit differential expression across all five datasets mentioned above. ns *p* > 0.05; *, *p* < 0.05; **, *p* < 0.01; ***, *p* < 0.001; ****, *p* < 0.0001.

To investigate the expression patterns of LILR family members across various human malignancies, we utilized the GSCA database to explore the differential expression of LILR family genes. As shown in Figure 2A, LILRA1, LILRA2, LILRA5, LILRB1, LILRB2, and LILRB3 are differentially expressed across several cancer types, including lung squamous cell carcinoma, lung adenocarcinoma, papillary renal cell carcinoma, and clear cell renal cell carcinoma, suggesting a potential pan-cancer role for LILR family genes. Specifically, for differential expression in ccRCC, we conducted differential analysis using expression data obtained from the TCGA-KIRC cohort. All 10 LILR family genes were significantly overexpressed in ccRCC samples. To enhance the robustness of this conclusion, we incorporated four datasets—GSE15641, GSE36895, GSE40435, and GSE53757—to validate the differential analysis results (Figure 2B–F). Ultimately, as depicted in Figure 2G, six genes were identified as differentially expressed genes (DEGs) with significant expression differences across the five datasets.

### 3.3. Identification of prognosis-related genes of the LILRs in ccRCC

**Figure 3.**
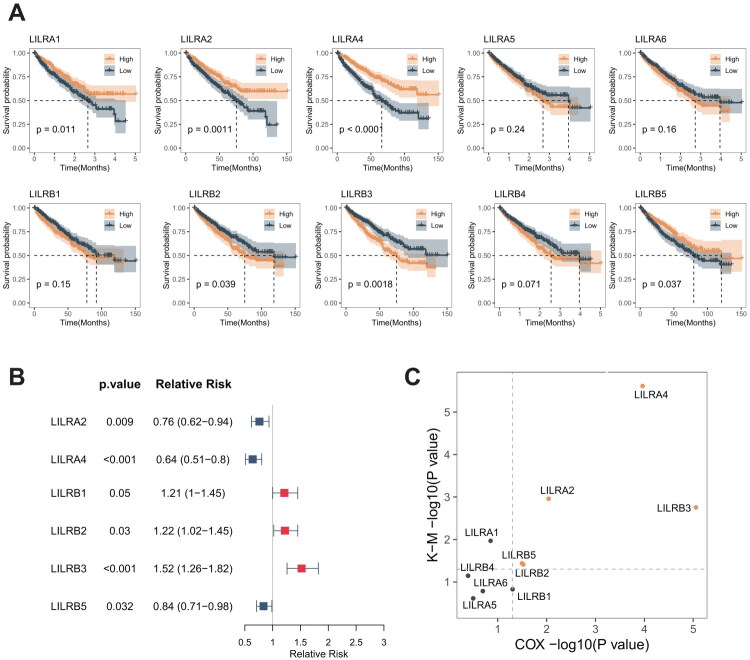
(A) Kaplan-Meier Curve analysis identifies the prognostic value of LILR family genes. (B) Univariate cox regression identifies clinical risk factors and protective factors. (C) Genes that exhibit prognostic value in both Kaplan-Meier curve analysis and univariate cox regression analysis.

Research on the prognostic implications of LILR family genes in ccRCC remains limited. Therefore, we employed Kaplan-Meier survival analysis and univariate Cox regression analysis to identify prognosis-related genes. The Kaplan-Meier survival analysis results indicated that LILRA1, LILRA2, LILRA4, LILRB2, LILRB3, and LILRB5 are associated with the prognosis of ccRCC (Figure 3A). Univariate Cox regression analysis revealed that LILRA2, LILRA4, and LILRB5 serve as protective factors for ccRCC prognosis, whereas LILRB1, LILRB2, and LILRB3 are risk factors (Figure 3B). In summary, LILRA2, LILRA4, LILRB2, LILRB3, and LILRB5 were identified as prognosis-related genes (Figure 3C).

### 3.4. Development and validation of LILRs-based prognostic signature

**Figure 4.**
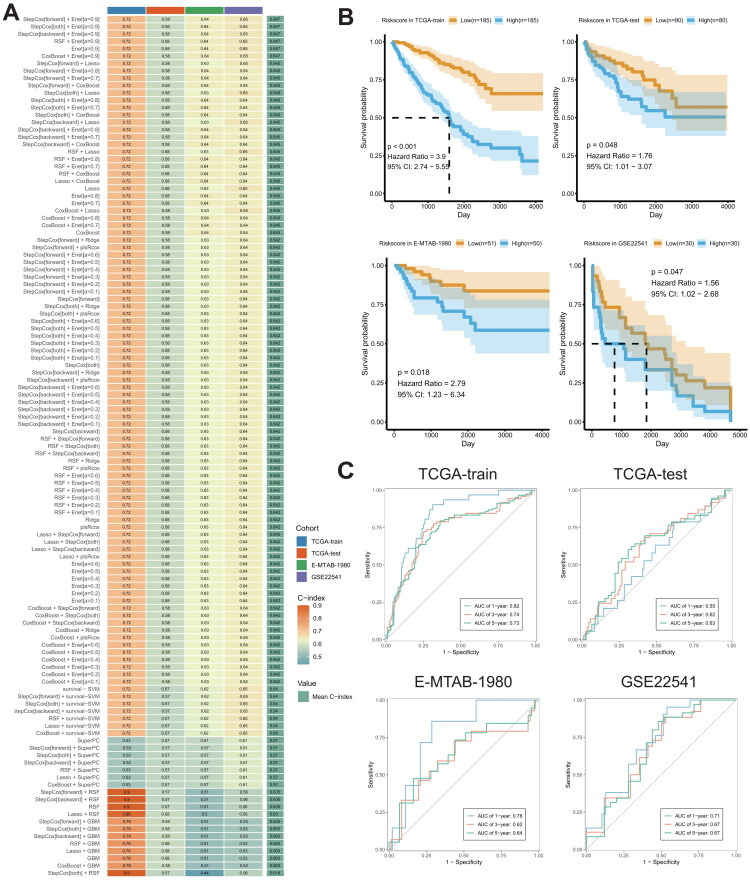
(A) C-Index computation for models derived from a comprehensive combination of 101 machine learning algorithms on LILR features, utilizing a 10-fold cross-validation framework. Datasets: TCGA training set, TCGA validation set, E-MTAB-1980, GSE22541. (B) Kaplan-meier survival curves depicting overall survival (OS) for patients with high and low LARS. (C) Time-dependent ROC curves illustrating 1-year, 3-year, and 5-year OS.

In the subsequent analysis, utilizing 10-fold cross-validation, we integrated ten machine learning algorithms, including RSF, Enet, stepwise Cox, CoxBoost, plsRcox, Lasso, Ridge, SuperPC, GBM, and survival-SVM, to construct robust LILR prognostic features based on core prognostic genes. Ultimately, the StepCox[forward] + Enet[*α* = 0.9] algorithm, which exhibited the highest C-index, was employed to develop a risk prediction model comprising LILRA2, LILRB2, LILRB3, and LILRB5 (Figure 4A). By calculating the median of the risk scores, we stratified ccRCC patients into high-risk and low-risk groups. Survival curve analysis indicated that low-risk group ccRCC patients had better prognoses across the TCGA training set, TCGA validation set, E-MTAB-1980, and GSE22541 datasets (Figure 4B). Time-dependent ROC curve analysis demonstrated the model’s high predictive accuracy (Figure 4C).

### 3.5. Analysis of the clinical relevance of the LILRs-based prognostic signature

**Figure 5.**
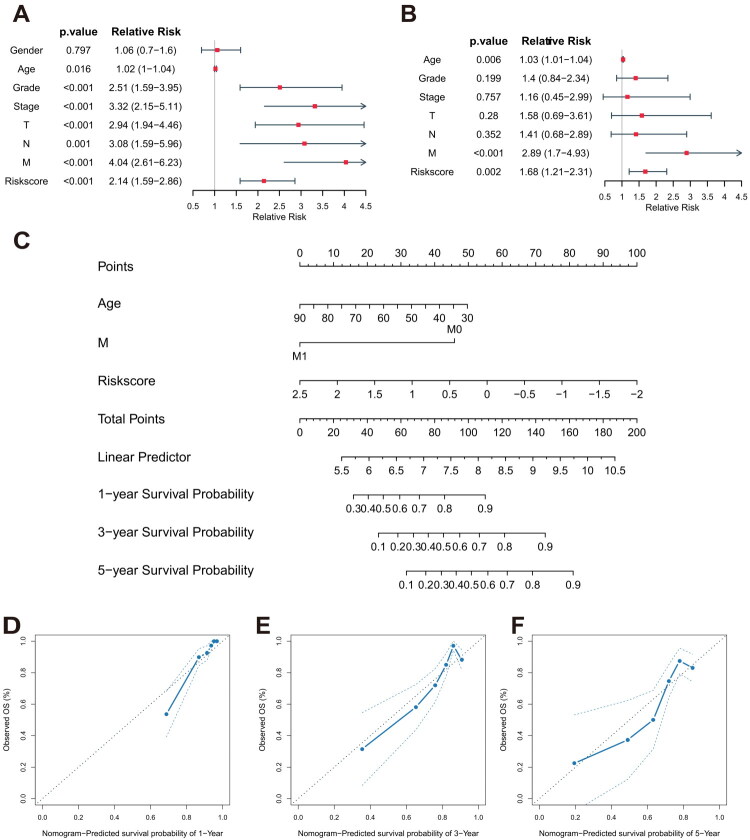
(A-B) Univariate and multivariate cox regression analyses identify independent risk factors affecting the prognosis of patients with ccRCC. (C) Prognostic nomogram constructed based on age, M stage, and risk score. (D-F) Calibration plots evaluating the accuracy of the prognostic nomogram in predicting 1-year, 3-year, and 5-year survival rate.

To explore the correlation between the LILRs-based prognostic signature and the clinicopathological characteristics of tumors, we selected 247 samples with complete clinicopathological data from the TCGA-KIRC cohort to investigate independent risk factors affecting the prognosis of ccRCC patients. Bar charts and box plots illustrate that patients with higher tumor stages, more advanced metastatic stages, and poorer nuclear grading exhibit elevated LARS levels (Supplementary Figure 2A–J). Through univariate and multivariate Cox regression analyses, we identified risk score (RR = 1.68, 95% CI [1.21, 2.31], *p* = 0.002), age (RR = 1.03, 95% CI [1.01, 1.04], *p* = 0.006), and M stage (RR = 2.89, 95% CI [1.7, 4.93], *p* < 0.001) as independent risk factors influencing ccRCC survival outcomes (Figure 5A, B). To visually present the correlation between independent risk factors and survival outcomes, a prognostic nomogram was constructed (Figure 5C). A calibration plot, a graphical tool used to demonstrate the accuracy of model-predicted probabilities, shows higher predictive reliability when actual points are closer to the ideal line (45-degree line). Calibration plot analysis indicated that the prognostic prediction model exhibited good reliability in predicting 1-year, 3-year, and 5-year survival outcomes for patients (Figure 5D–F).

### 3.6. Functional pathway analysis of the LILRs-based prognostic signature

**Figure 6.**
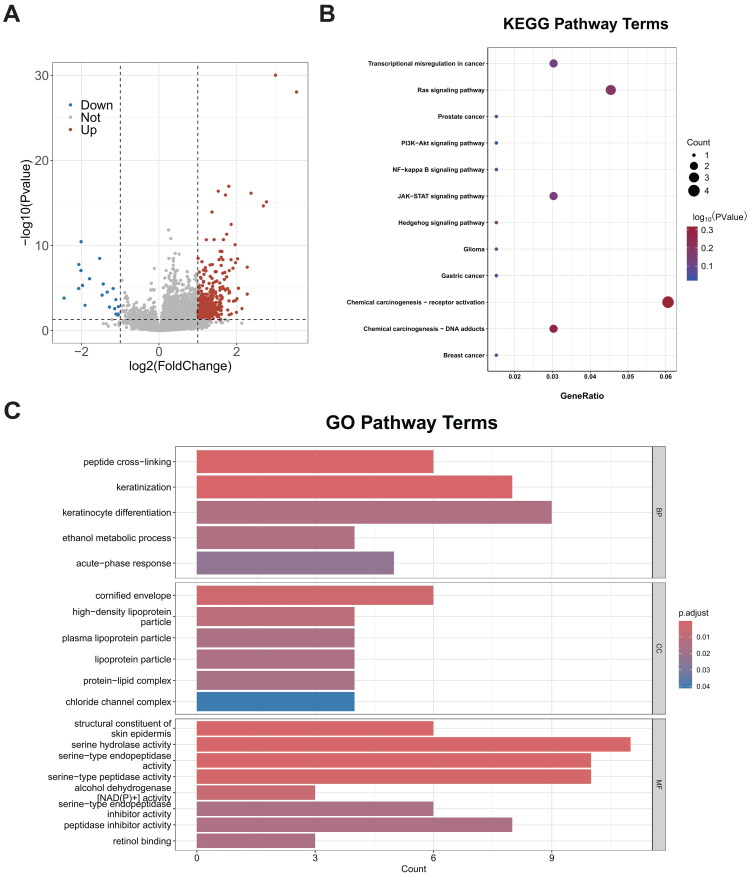
(A) Differential analysis identifies genes that are distinct between high and low LARS groups. (B) KEGG enrichment analysis reveals pathways significantly enriched in the high LARS group. (C) GO enrichment analysis demonstrates pathways significantly enriched in the high LARS group within biological process (BP), cellular component (CC), and molecular function (MF).

To explore the downstream functional pathways associated with the LILRs-based prognostic signature, we conducted enrichment analysis. As shown in Figure 6A, we analyzed genes that exhibited significant differences between the high LARS and low LARS groups (|log2FC| > 1 and *p* < 0.05). KEGG analysis reveals that LARS is associated with the progression of cancers such as gastric, breast, and prostate cancer. It may also play a role in regulating critical cancer pathways, including the PI3K-Akt signaling pathway and the JAK-STAT signaling pathway (Figure 6B). GO enrichment analysis indicates that LARS may be involved in various protein regulatory processes in the human body, such as peptide cross-linking, protein-lipid complex formation, and endopeptidase regulator activity (Figure 6C).

### 3.7. Immuno-infiltration correlation analysis

**Figure 7.**
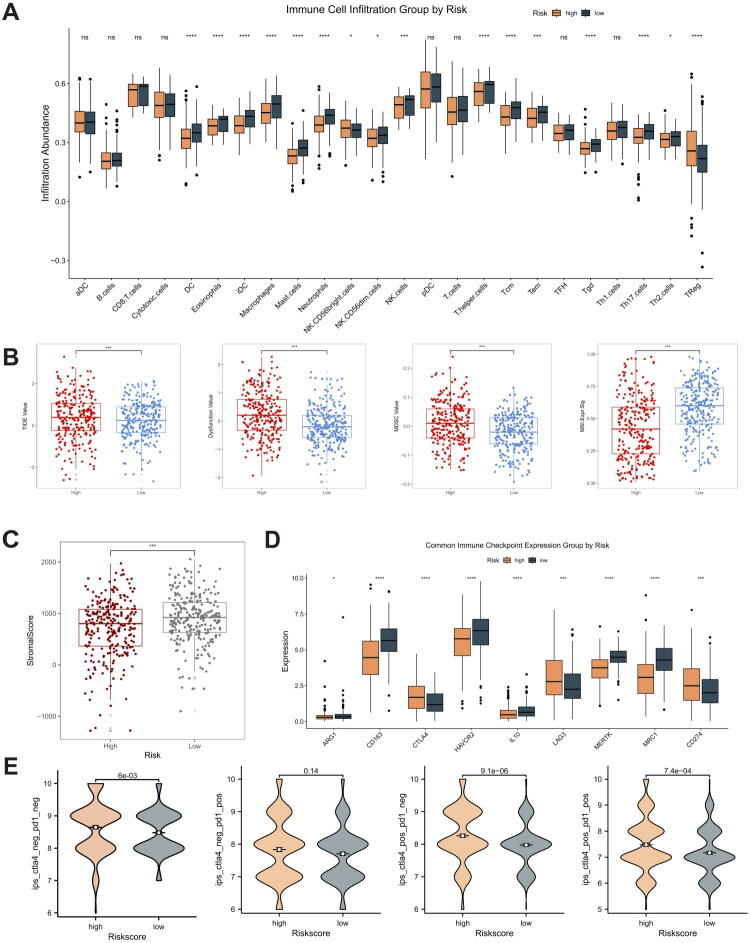
(A) The infiltration abundance of various immune cells between high and low LARS groups. (B) The differences in TIDE analysis between high and low LARS groups. (C) The stromal score between high and low LARS groups. (D) The differences in immune checkpoint expression levels between the high and low LARS groups. (E) Predicting immunotherapy sensitivity using IPS score.

Previous enrichment analysis suggests that LILR may affect the prognosis of ccRCC patients by participating in immune-related pathways. To further explore the role of LARS in the tumor immune microenvironment (TIME), we conducted single-sample gene set enrichment analysis (ssGSEA). The results indicate that in cohorts with lower LARS, immune cells such as dendritic cells, macrophages, and NK cells are significantly increased, while Treg cells are significantly elevated in cohorts with higher LARS (Figure 7A). TIDE (Tumor Immune Dysfunction and Exclusion) analysis is a tool used to evaluate the effectiveness of tumor immunotherapy. By analyzing immune evasion mechanisms within the tumor microenvironment, it helps predict patient responses to immune checkpoint inhibitors, such as PD-1 or CTLA-4 inhibitors. Generally, a higher TIDE score indicates more significant immune evasion mechanisms in the tumor. TIDE analysis further reveals a correlation between increased LARS expression and higher TIDE scores, as well as increased immune dysfunction scores (Figure 7B). Additionally, ESTIMATE analysis shows a connection between higher LARS expression and stromal scores (Figure 7C). These findings suggest that patients with higher LARS exhibit greater immune cell infiltration, accompanied by decreased immune cell function. This phenomenon may be complexly linked to mechanisms in ccRCC that inhibit T cell activation and promote tumor immune evasion. Immune checkpoint inhibitors (ICIs) play a crucial role in tumor treatment for ccRCC, so we examined the relationship between LARS and classic immune checkpoint expression levels. Most immune checkpoint expression levels are lower in patients with high LARS, but CD274 (PD-L1) and CTLA4 expression levels are higher (Figure 7D). Targeting these upregulated immune checkpoint genes may benefit patients with this tumor subtype. The TCIA database provides tools to predict immunotherapy responsiveness based on immunogenicity scores (IPS). We observed that the high LARS group had significantly higher IPS in different immune subgroups, including CTLA-4 and PD-1 negative, CTLA-4 positive and PD-1 negative, and CTLA-4 positive and PD-1 positive, compared to the low LARS group (*p* < 0.05; Figure 7E). This suggests that patients in the high LARS group may respond better to PD-L1 and CTLA4-based immunotherapy.

### 3.8. Putative small molecules correlated with risk score

**Figure 8.**
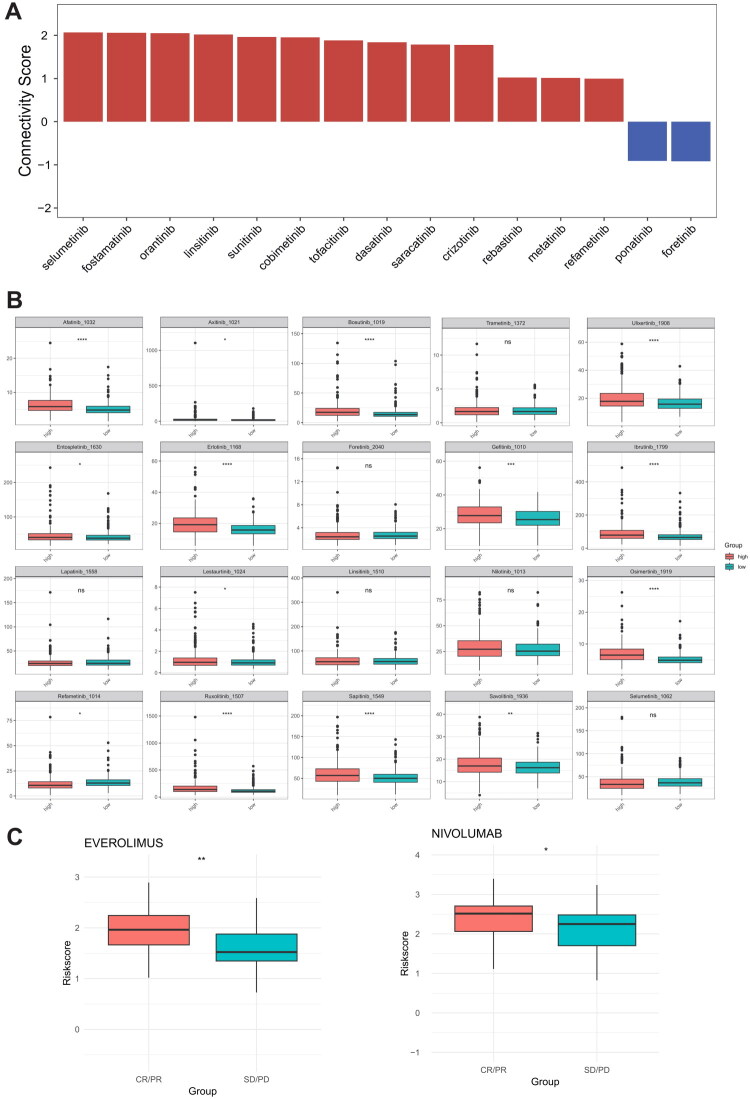
(A) Association between TKIs and risk scores based on the CMAP database; (B) Differential analysis of 20 TKIs from the GDSC database between high- and low-risk groups; (C) Differential analysis of nivolumab and everolimus between high- and low-risk groups.

To further investigate the association between tyrosine kinase inhibitors (TKIs) and risk scores, we inputted 150 upregulated/downregulated differentially expressed genes (DEGs) from the two groups into the CMap database to obtain the normalized connectivity scores reflecting the correlation between TKIs and risk scores (Figure 8A). The bar chart in the figure illustrates the TKIs that exhibit inhibitory effects in both groups: The red bars indicate potent inhibitors effective against cancer cells from low-risk patients, whereas the blue bars indicate significant inhibitors targeting cancer cells from high-risk patients. Additionally, analysis of 20 tyrosine kinase inhibitors from the GDSC database revealed that 14 drugs, including Afatinib, demonstrated enhanced sensitivity in high-risk patients, whereas Refametinib exhibited greater sensitivity in the low-risk patient group (Figure 8B). Meanwhile, we analyzed the data provided by Braun et al. from CheckMate-009, CheckMate-010, and CheckMate-025 studies, and found that patients with ccRCC who received Nivolumab and Everolimus and achieved CR (complete response) or PR (partial response) outcomes exhibited higher risk scores (Figure 8C).

### 3.9. Identification and expression level analysis of hub genes

**Figure 9.**
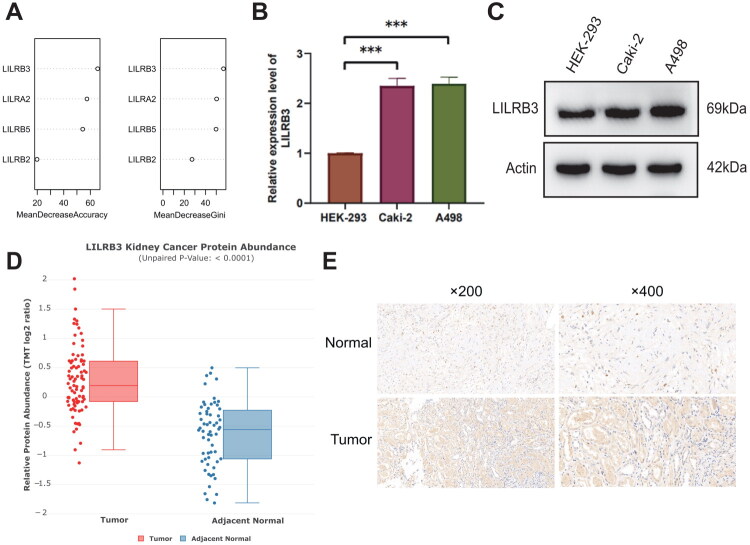
(A) The random Forest method analyzes the mean decrease in accuracy and the mean decrease in gini index for each gene in the prognostic model. (B) RT-qPCR measures the mRNA expression levels of LILRB3 in human normal renal cell lines and ccRCC cells. (C) Western blot analyzes the protein expression levels of LILRB3 in human normal renal cell lines and ccRCC cells. (D) Analysis of CPTAC data reveals differences in LILRB3 abundance levels between renal tumors and normal kidney tissue. (E) Immunohistochemistry evaluates the expression of LILRB3 in ccRCC and adjacent normal tissues. (*p < 0.05; **p < 0.01; ***p < 0.001).

To delve deeper into the genes playing pivotal roles in the predictive model, we employed the random forest algorithm for selection. We discovered that the average Gini index reduction for LILRB3 was 65.57, with an average accuracy decrease of 56.86 (Figure 9A). These metrics indicate that LILRB3 makes the most significant contribution among the four genes constituting the prognostic model. To validate the bioinformatics analysis, measurements revealed that LILRB3 expression levels were markedly elevated in Caki-2 and A498 cell lines compared to the human embryonic kidney cell line HEK-293 (Figure 9B, C). CPTAC analysis indicates that LILRB3 is significantly more abundant in renal tumors than in normal kidney tissue (Figure 9D). Furthermore, we assessed LILRB3 expression in ccRCC tissues and adjacent non-cancerous tissues using immunohistochemistry (IHC). The IHC results demonstrated that LILRB3 exhibited high staining intensity in ccRCC tissues, primarily localized to the cell membrane, while showing weakly positive or negative expression in adjacent non-cancerous tissues (Figure 9E). Further analysis of the association between LILRB3 and clinical factors revealed that increased LILRB3 expression was correlated with higher TNM stages and advanced stages, with no significant differences observed concerning age and gender (Supplementary Figure 4A–F).

### 3.10. The impact of LILRB3 knockdown on the proliferation and migration of clear cell renal cell carcinoma cells

**Figure 10.**
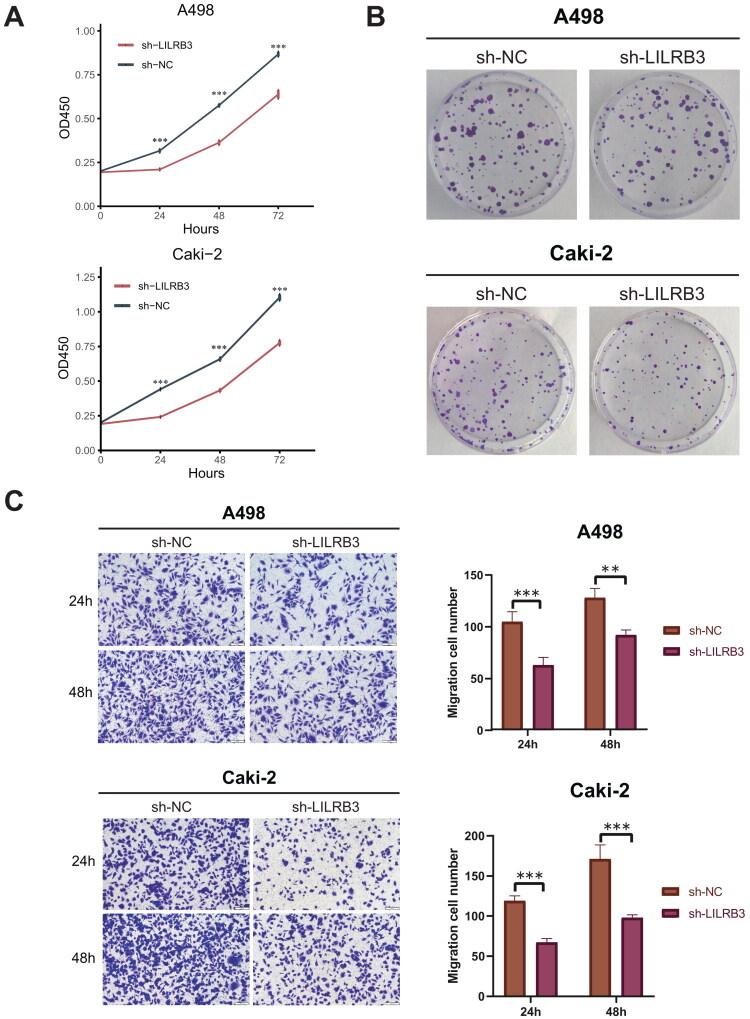
(A) The CCK-8 proliferation assay confirms that silencing LILRB3 significantly inhibits the proliferative capacity of A498 and caki-2 cell lines. (B) The colony formation assay demonstrates the impact of LILRB3 silencing on the clonogenic ability of A498 and caki-2 cell lines. (C) Silencing LILRB3 significantly suppresses the migratory capacity of A498 and caki-2 cell lines. (magnification: 100×, *p < 0.05; **p < 0.01; ***p < 0.001).

To investigate the impact of LILRB3 gene silencing on the proliferation and migration abilities of ccRCC cells, we established LILRB3 knockdown renal cancer cell lines. In the three knockdown groups, shLILRB3-1, shLILRB3-2, and shLILRB3-3, the expression levels of LILRB3 were significantly reduced compared to the control group sh-NC, demonstrating a clear transfection effect (*p* < 0.05). Furthermore, Western Blot results corroborated this finding, showing a marked decrease in LILRB3 expression levels in these knockdown groups compared to the control group (Supplementary Figure 3A–D). Based on these results, we selected shLILRB3-1 for further study in subsequent experiments. Initially, using the CCK-8 assay, we observed a significant reduction in cell proliferation in the Caki-2 and A498 cell lines following LILRB3 gene silencing, compared to the control group (Figure 10A). Additionally, the plate colony formation assay further confirmed the inhibitory effect of LILRB3 gene silencing on the proliferation capacity of ccRCC cells (Figure 10B). The results of the Transwell assay indicated that the migration ability of ccRCC cells with LILRB3 gene silencing was significantly reduced compared to the control group (Figure 10C). Collectively, these experimental results demonstrate that silencing the LILRB3 gene can significantly inhibit the proliferation and migration capabilities of ccRCC cells.

### 3.11. Knockdown of LILRB3 involves downregulation of the PI3K/AKT/mTOR signaling pathway and inhibition of PD-L1 expression

**Figure 11.**
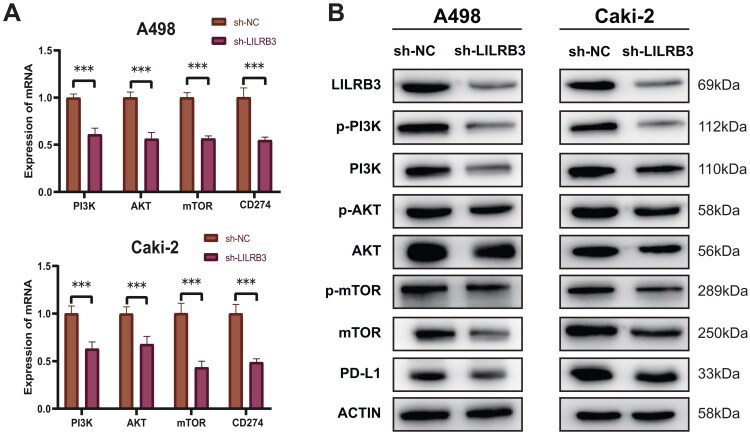
(A) RT-qPCR analysis of the expression of key genes in the PI3K/AKT/mTOR pathway and PD-L1 (CD274) in A498 and caki-2 cells after transfection with sh-LILRB3. (B) Western blot analysis of the expression of key proteins in the PI3K/AKT/mTOR pathway and PD-L1.

To investigate the potential mechanisms by which LILRB3 facilitates the progression of ccRCC, we categorized the samples based on the median expression level of LILRB3 and conducted enrichment analyses. KEGG analysis revealed that in samples with elevated LILRB3 expression, the PI3K-Akt signaling pathway, mTOR signaling pathway, PD-L1-related pathway, and immune-related pathways were significantly enriched (Supplementary Figure 5A). As illustrated in Supplementary Figure 5B, further GSEA analysis indicated a higher enrichment score for the PI3KCI signaling pathway in samples with increased LILRB3 expression (NES = 2.01, P.adj < 0.001, FDR < 0.001). Pearson correlation analysis demonstrated a significant positive correlation between the expression of LILRB3 and the PI3K subgroup member PIK3CG and mTOR (Supplementary Figure 5C, D). To explore the potential link between LILRB3 and this signaling pathway, we employed qPCR and Western Blot techniques to examine the expression of PI3K/AKT/mTOR pathway and PD-L1 at mRNA and protein levels in ccRCC cells. Figure 11A shows that in LILRB3-knockdown ccRCC cells, the expression levels of genes related to the PI3K/AKT/mTOR pathway and CD274 were downregulated. As depicted in Figure 11B, compared to cells transfected with control vectors, ccRCC cells with reduced LILRB3 expression exhibited significantly decreased protein levels of PI3K, p-PI3K, AKT, p-AKT, mTOR, p-mTOR, and PD-L1. This finding suggests that LILRB3 may regulate the PI3K/AKT/mTOR signaling pathway, with its expression changes directly affecting the activity of key proteins within the pathway. LILRB3 appears to exert a regulatory effect on PD-L1 expression in ccRCC, potentially facilitating tumor cells’ evasion of immune surveillance.

## 4. Discussion

Although many patients with early-stage renal cell carcinoma still have the opportunity to undergo curative surgical intervention, the five-year survival rate for those in locally advanced or metastatic stages remains disheartening [2]. Moreover, individuals exhibiting certain molecular characteristics may experience a heightened risk of postoperative recurrence [35]. Given the variability in prognosis among these renal cancer patients, precise risk assessment is crucial for identifying which individuals might benefit from more intensive initial treatment, closer surveillance, or adjuvant therapy. In the realm of malignant tumor treatment, the integration of novel biomarkers into the selection process for immunotherapeutic drugs has been adopted in clinical practice. Recent studies suggest that LILR-targeted therapies represent a promising avenue in cancer treatment, garnering significant attention due to their pivotal role in tumor progression and immune modulation [4]. However, the prognostic and therapeutic implications of LILRs in clear cell renal carcinoma remain to be further elucidated.

Given the pivotal role of polygenic traits in the risk stratification of renal cell carcinoma, we conducted a comprehensive multi-database analysis to examine the differential expression of LILR family genes across pan-cancer and ccRCC samples. Utilizing data from 530 ccRCC samples in the TCGA database, we constructed a risk prediction model to elucidate the prognostic significance of LILRs for ccRCC patients. The findings indicate that a risk scoring model composed of LILRA2, LILRB2, LILRB3, and LILRB5 can effectively distinguish patients with varying prognoses and predict long-term survival rates for ccRCC patients. This conclusion has been validated in 101 patient samples from the ArrayExpress database as well as in GSE22541.

Additionally, we incorporated the clinicopathological characteristics of ccRCC tumors into our analysis, employing univariate and multivariate Cox regression analyses to identify independent risk factors impacting patient prognosis. Based on these factors, a prognostic nomogram was developed to visually assess the influence of independent risk factors on patient survival outcomes. Considering the extensive role of LILRs in human immunity, we further evaluated differences in immune cell infiltration levels between high- and low-risk groups using ssGSEA and ESTIMATE analyses. The results revealed significant enrichment of TME-related immune cells, including CD4+ T cells, CD8+ T cells, aDCs, and MDSCs, in the high-risk group. To further validate the potential role of the LILR family in the progression of ccRCC, we applied random forest algorithms and PPI interaction network analysis based on the genes in the risk prediction model to identify LILRB3 as a core gene influencing the prognosis of ccRCC patients.

Compared to the LILRA subfamily, the LILRB subfamily has garnered more extensive attention in cancer research due to its role in mediating immunosuppressive signaling. Compelling evidence suggests that LILRB is implicated in tumorigenesis, as well as in tumor immune evasion and progression [36–40]. LILRB is typically overexpressed in cells associated with immunosuppression, such as immunosuppressive M2 macrophages and tolerogenic dendritic cells (tol-DCs). In the context of innate immunity, the overexpression of LILRB can inhibit the phagocytic activity of neutrophils and macrophages. Regarding adaptive immunity, LILRB can suppress B cell responses through both T cell-dependent and independent mechanisms [4,38, 39,41,42]. Additionally, during dendritic cell development, LILRB engagement enhances tolerance by raising the activation threshold, promoting the release of anti-inflammatory cytokines, activating CD4+ helper T cells, and converting them into regulatory T cells (Tregs). Recent studies have revealed a close association between LILRB and the progression of hematologic malignancies [4,43,44]. Meanwhile, the LILRB subfamily is highly expressed in solid tumors, including breast cancer, prostate cancer, gastric cancer, colorectal cancer, pancreatic cancer, and lung cancer, and is directly linked to poorer clinical staging, diminished therapeutic responses, and reduced overall survival rates [40,42,45]. At present, various therapeutic strategies targeting LILRB are undergoing preclinical studies and clinical trials [41,43, 46–48].

LILRB3, also referred to as ILT5, CD85a, or LIR3, is an inhibitory molecule within the LILR family that contains four cytoplasmic ITIM motifs. These motifs enable the recruitment of tyrosine phosphatases SHP1, SHP2, and inositol phosphatase SHIP, thereby facilitating its immunosuppressive function [47]. LILRB3 plays a crucial role in regulating tumor growth and proliferation. In acute myeloid leukemia, activation of LILRB3 leads to the formation of the LILRB3-TRAF2 complex, which recruits cFLIP, subsequently activating NF-κB. NF-κB can initiate the expression of multiple target genes that promote cell survival and inhibit apoptosis, such as Bcl-2, c-IAPs, and FLIP. Through the activation of these genes, NF-κB supports cell viability, providing essential conditions for cell proliferation. In normal monocytes, LILRB3 assumes a dual role in NF-κB signaling, depending on the duration of activation stimuli: inflammatory stimuli briefly activate LILRB3, promoting NF-κB activation; whereas prolonged activation of LILRB3 recruits phosphatases SHP-1 and SHP-2, interacting with the LILRB3-TRAF2 complex, disrupting TRAF2 degradation mediated by the zinc finger protein A20. In malignant monocytes, the balance between LILRB3-mediated NF-κB activation and inhibition is altered, with prolonged activation failing to suppress NF-κB signaling, resulting in sustained pathway activation [49]. Research by Zhuang et al. indicates that LILRB3 is predominantly expressed on monocyte-derived tumor-associated macrophages (Mo-TAMs) in gliomas, with high LILRB3 expression serving as an independent negative prognostic factor across various glioma grades [8]. The interaction between glioblastoma multiforme (GBM) cell states and macrophages may play a pivotal role in modulating the tumor immune environment. Studies suggest that macrophages can induce GBM cells to transition into mesenchymal-like states (MES-like states), which endow the cells with mesenchymal-like characteristics, such as enhanced migratory capacity, invasiveness, and resistance to therapy, thereby significantly impacting tumor progression and immune evasion mechanisms.

Our objective is to investigate the role of LILRB3 in the biological behavior of ccRCC. Initially, we validated the aberrant expression of LILRB3 in ccRCC at both transcriptomic and proteomic levels. Subsequently, we established LILRB3-knockdown ccRCC cell lines and assessed the impact of LILRB3 on cell proliferation using CCK-8 and colony formation assays. The experimental results demonstrated that the downregulation of LILRB3 significantly inhibited the proliferative capacity of ccRCC cells, thereby confirming the promotive effect of LILRB3 on ccRCC cell proliferation *in vitro*. Furthermore, through Transwell migration assays, we observed a marked reduction in the migratory ability of cells with downregulated LILRB3 expression compared to the control group. Concurrently, we employed flow cytometry and Western Blot techniques to delve into the molecular mechanisms of LILRB3 in ccRCC. The experimental results revealed that reducing LILRB3 expression in ccRCC cells *via* RNA interference significantly modulates the expression of key proteins in the PI3K/AKT/mTOR signaling pathway and the immune checkpoint molecule PD-L1. These findings highlight the complex role of LILRB3 in ccRCC. On one hand, LILRB3 may promote tumor cell proliferation and metastasis by activating the PI3K/AKT/mTOR signaling pathway; on the other hand, the upregulation of PD-L1 expression by LILRB3 might contribute to establishing an immunosuppressive tumor microenvironment, enabling tumor cells to evade immune recognition and clearance, thereby facilitating further tumor progression and deterioration.These findings indicate that LILRB3 plays a pivotal role in the functionality of ccRCC cells, and modulating its expression levels may represent a potential therapeutic strategy for ccRCC.

## 5. Limitations

However, our study still has several important limitations. Firstly, the risk prediction model based on the LILR family primarily relies on retrospective data from public databases. Although it has demonstrated good prognostic value, further validation in larger, multicenter prospective cohorts is necessary to enhance its generalizability and clinical applicability. Secondly, the sample size for immunohistochemical validation of LILRB3 expression differences between tumor tissues and adjacent normal tissues was limited, which to some extent affects the statistical power and generalizability of our conclusions. Therefore, future studies should incorporate larger patient cohorts for more extensive validation. In addition, there are certain limitations regarding cell models and functional experiments in this study. We only selected two renal cancer cell lines for *in vitro* experiments, without including other classic renal cancer cell lines such as 786-O and 769-P, nor did we conduct a broader range of functional assays such as Transwell migration, wound healing, cell cycle, or apoptosis analysis. This somewhat restricts our in-depth understanding of the biological role of LILRB3 in tumors. Although we analyzed the correlation between LILR expression and potential therapeutic sensitivities—such as to tyrosine kinase inhibitors—using tools like CMap and CellMiner, future studies should further expand drug-gene interaction analysis with methods such as molecular docking to clarify its translational medical value. Furthermore, the specific action sites and downstream signaling pathways of LILRB3 have not yet been thoroughly elucidated. The immune regulatory effects induced by LILRB3 activation and its mechanisms within the tumor microenvironment require further clarification, which will necessitate the use of animal models and more mechanistic studies. In the future, we plan to expand tissue validation in larger patient cohorts, enrich both *in vivo* and *in vitro* functional experiments, and integrate multi-omics and immunotherapy cohort data to further validate and extend our findings. Ultimately, this will provide a theoretical basis for a deeper understanding of the immunological and clinical significance of LILRB3 in renal cancer and the development of novel immunotherapeutic strategies.

## 6. Conclusions

We constructed a risk prediction model based on the LILR family genes, which consistently demonstrated significant prognostic utility in evaluating survival outcomes, clinicopathological features, immune infiltration, and the efficacy of immunotherapy in ccRCC patients. We further identified LILRB3 as a key gene and demonstrated its differential expression in ccRCC and its impact on the phenotype of ccRCC cells. These findings reveal the potential of the LILR family to provide precise therapeutic strategies for ccRCC patients.

## Supplementary Material

Supplemental Material

## Data Availability

The transcriptomic data, clinical information, and somatic mutation data utilized in this study are freely available from the TCGA database (https://portal.gdc.cancer.gov/projects/TCGA-KIRC/). External validation data, E-MTAB-1980, is accessible from the ArrayExpress database (https://www.ebi.ac.uk/arrayexpress/). Access to the GSE40435 dataset is available *via* the following link: https://www.ncbi.nlm.nih.gov/geo/query/acc.cgi?acc=GSE40435. The TIDE analysis was performed using publicly accessible data, which can be obtained from the TIDE website (http://tide.dfci.harvard.edu). The analyzed experimental data pertaining to human subjects are available upon reasonable request to the corresponding author.
