# Supplementary material for: Comprehensive analysis of the leukocyte immunoglobulin-like receptor family in clear cell renal cell carcinoma
Source: Ann Med. 2025 Aug 22;57(1):2546684. doi: 10.1080/07853890.2025.2546684 (PMC12377148; doi:10.1080/07853890.2025.2546684)
Supplement: Supplemental Material [file IANN_A_2546684_SM0491.zip › suppl_data/Supplementary Table legends.docx]

**Supplementary Table 1.** Genes related to the LILR family

**Supplementary Table 2.** Primer sequences of LILRB3 and Actin

**Supplementary Fig 1**

(A) The Actin and LILRB3 bands corresponding to Figure 8C. (B) The Actin and LILRB3 bands corresponding to Supplementary Figure 3B. (C) The Actin and LILRB3 bands corresponding to Supplementary Figure 3D. (D) The key proteins in the PI3K/AKT/mTOR pathway and PD-L1 bands in A498 corresponding to Figure 10B (E) The key proteins in the PI3K/AKT/mTOR pathway and PD-L1 bands in Caki-2 corresponding to Figure 10B.

**Supplementary Fig 2**

(A-J) Bar charts and box plots illustrate that patients with higher tumor stages, more advanced metastatic stages, and poorer nuclear grading exhibit elevated LARS levels.

**Supplementary Fig 3**

(A-D) In A498 and Caki-2 cells, the expression level of LILRB3 was significantly reduced in the gene knockdown group compared to the control group.

**Supplementary Fig 4**

(A-F) Differences in LILRB3 expression between different clinicopathological stages.

**Supplementary Fig 5**

(A) GO enrichment analysis between high and low expression groups of LILRB3. (B) GSEA enrichment analysis between high and low expression groups of LILRB3. (C-D) Expression correlation of LILRB3 with PIK3CG and MTOR.
