# Supplementary figures and images for: Comprehensive analysis of the leukocyte immunoglobulin-like receptor family in clear cell renal cell carcinoma
Source: Ann Med. 2025 Aug 22;57(1):2546684. doi: 10.1080/07853890.2025.2546684 (PMC12377148; doi:10.1080/07853890.2025.2546684)

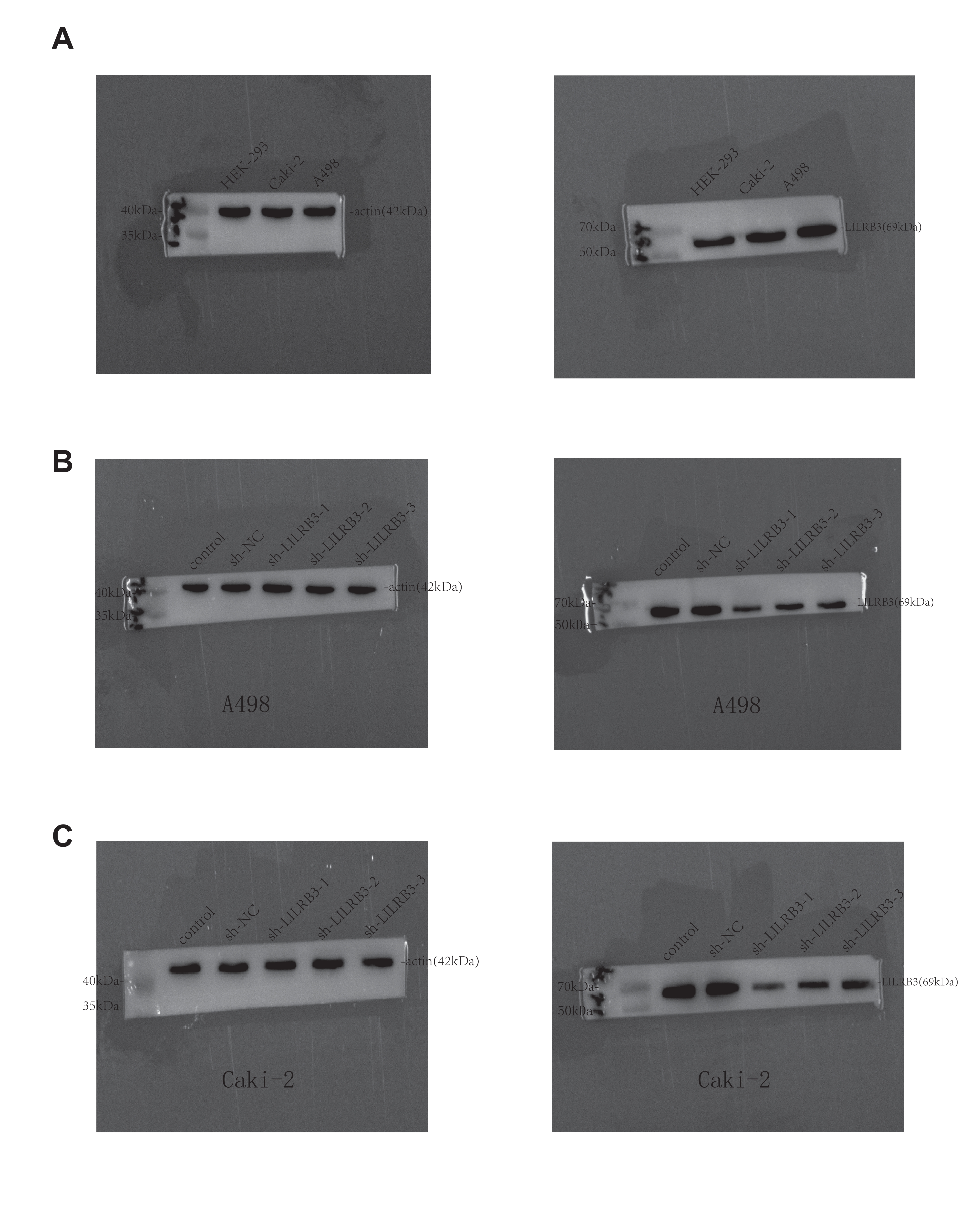

Supplement: Supplemental Material [file IANN_A_2546684_SM0491.zip › suppl_data/Supplementary Figure 1ABC.jpg]

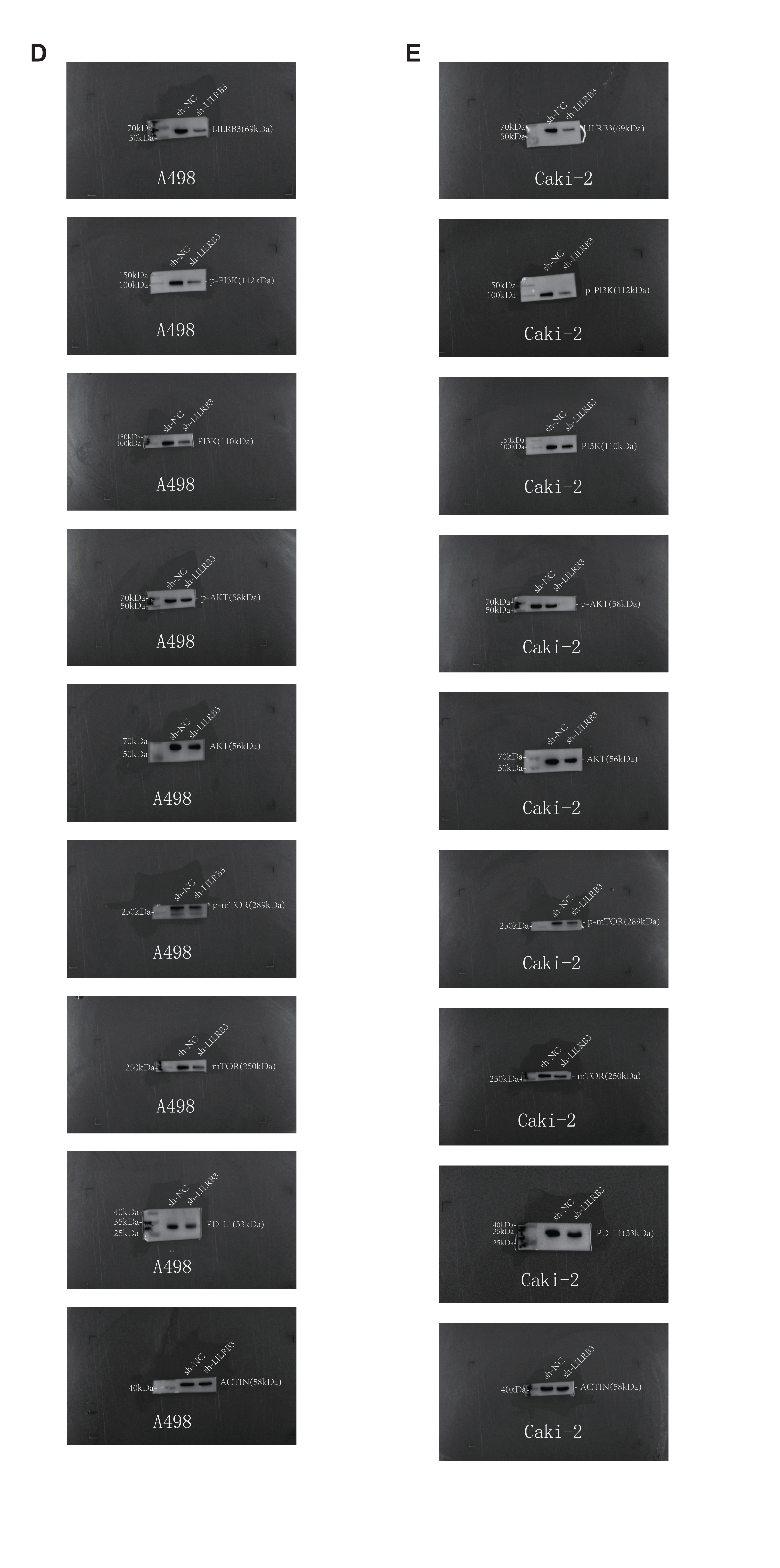

Supplement: Supplemental Material [file IANN_A_2546684_SM0491.zip › suppl_data/Supplementary Figure 1DE.jpg]

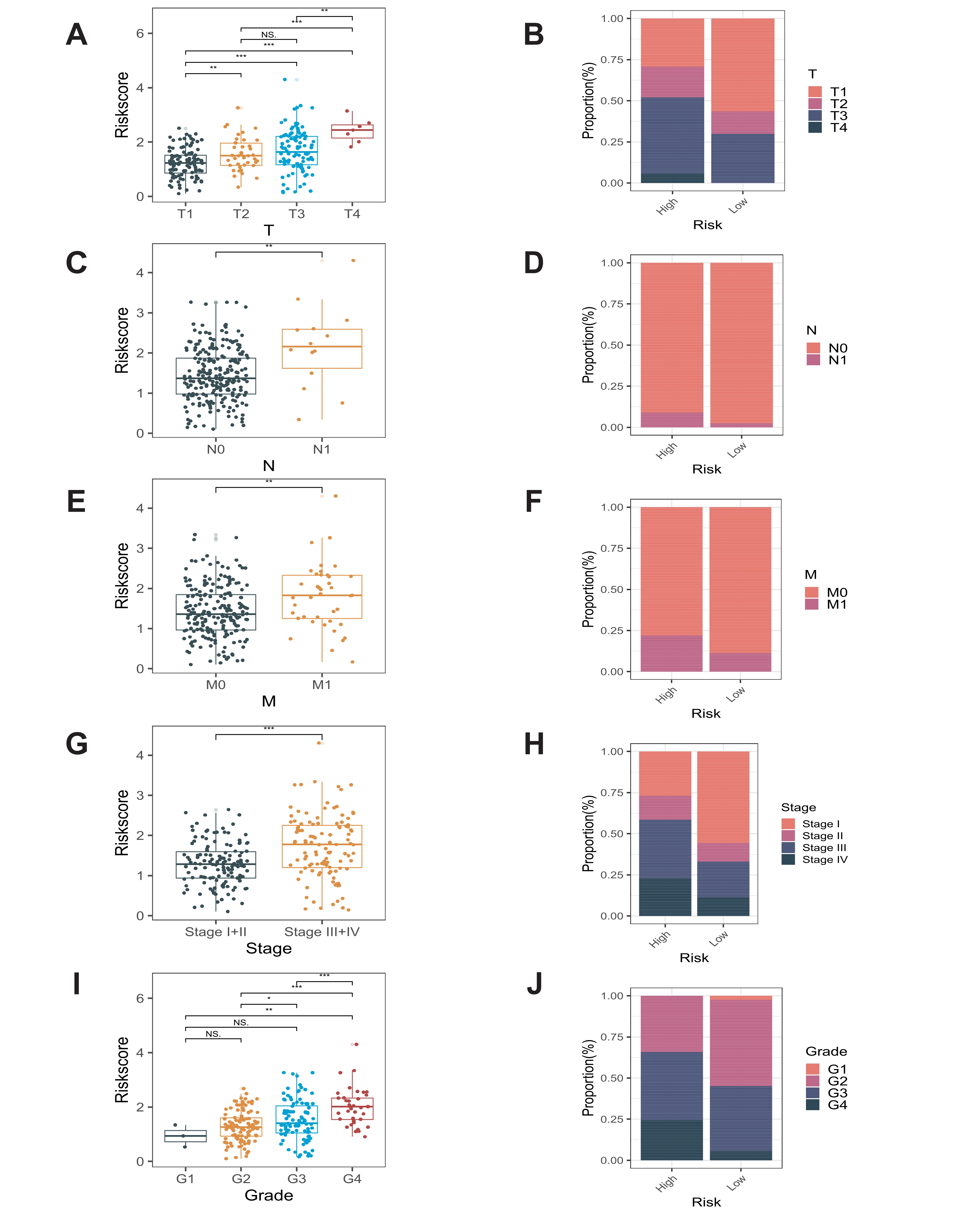

Supplement: Supplemental Material [file IANN_A_2546684_SM0491.zip › suppl_data/Supplementary Figure 2.jpg]

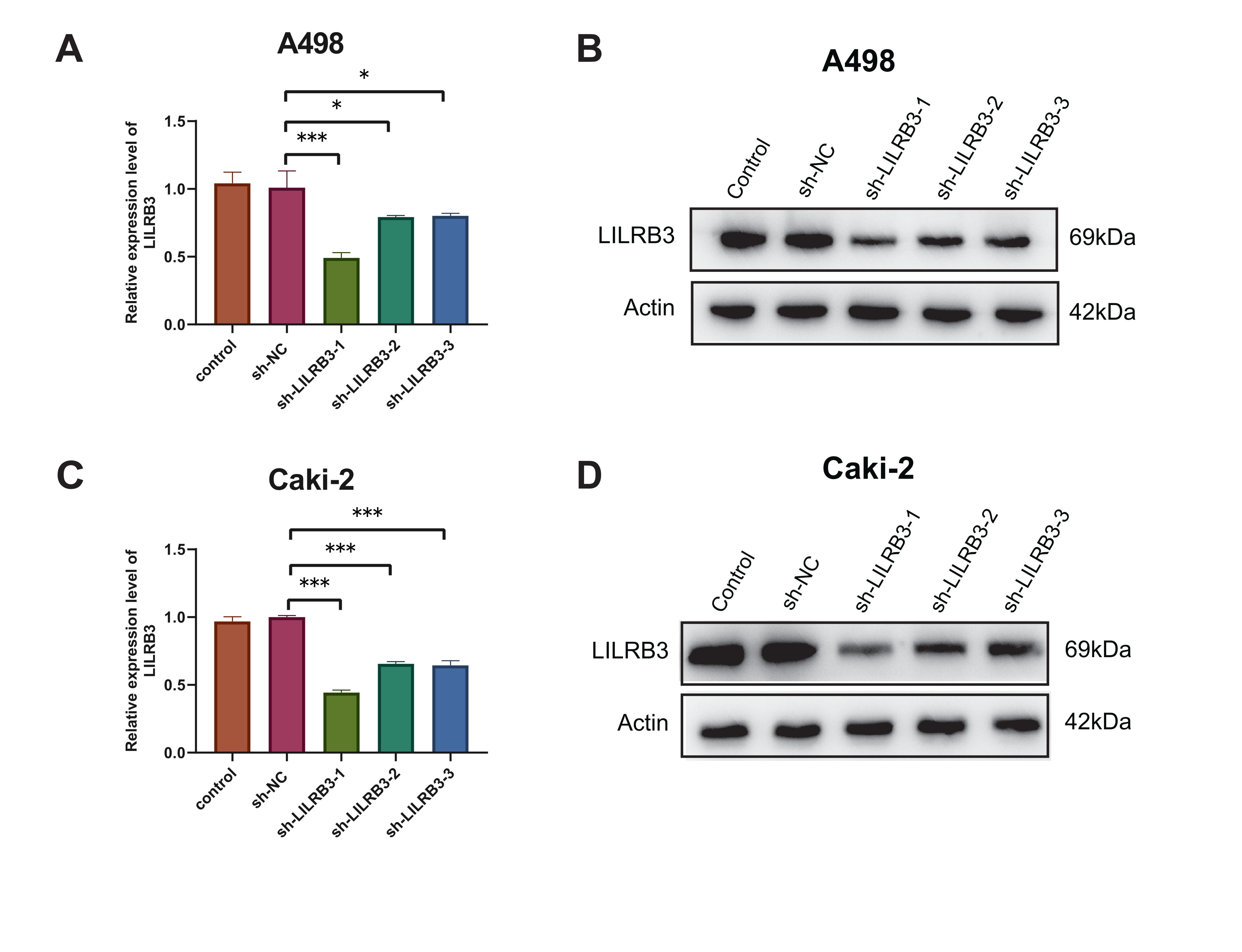

Supplement: Supplemental Material [file IANN_A_2546684_SM0491.zip › suppl_data/Supplementary Figure 3.jpg]

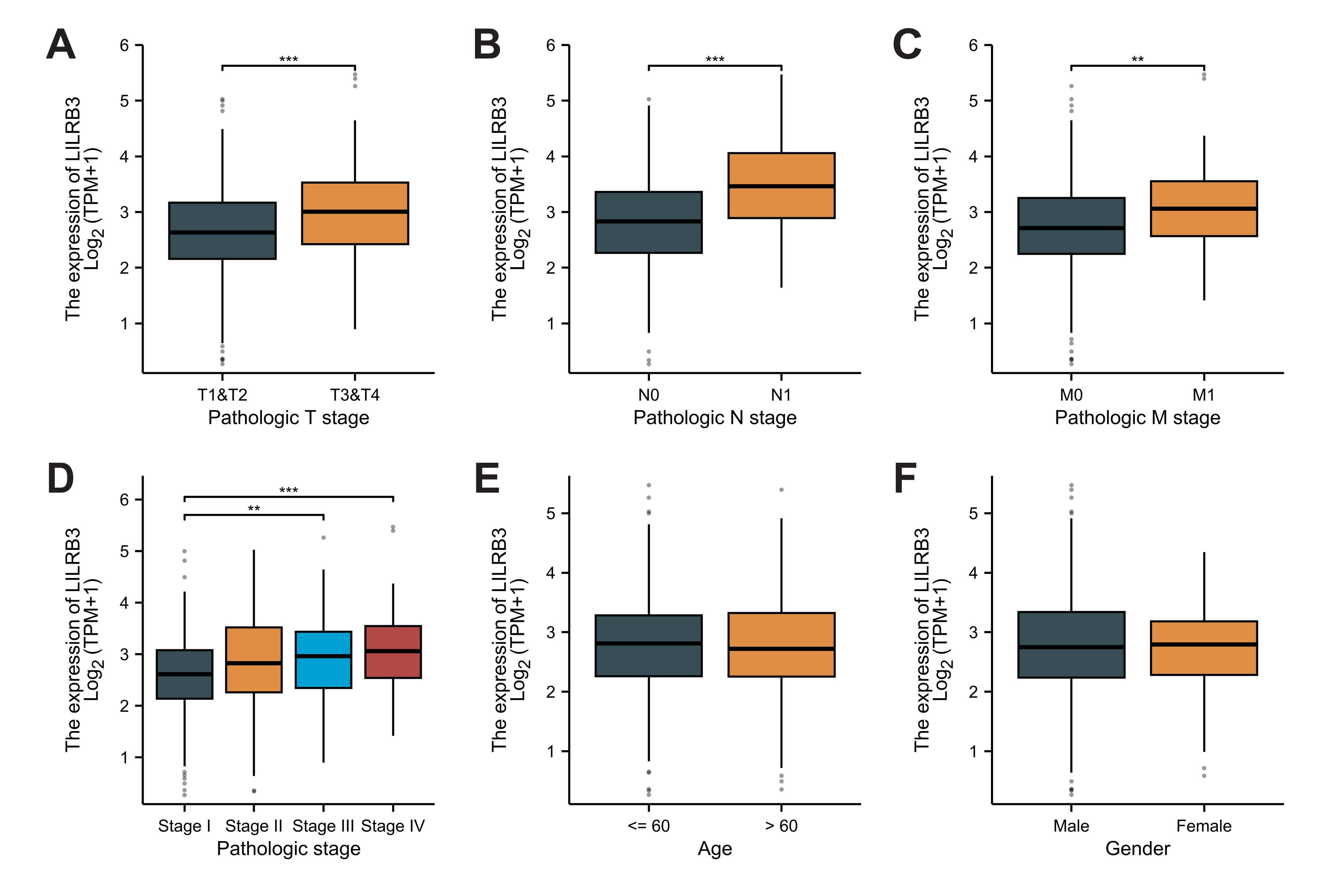

Supplement: Supplemental Material [file IANN_A_2546684_SM0491.zip › suppl_data/Supplementary Figure 4.jpg]

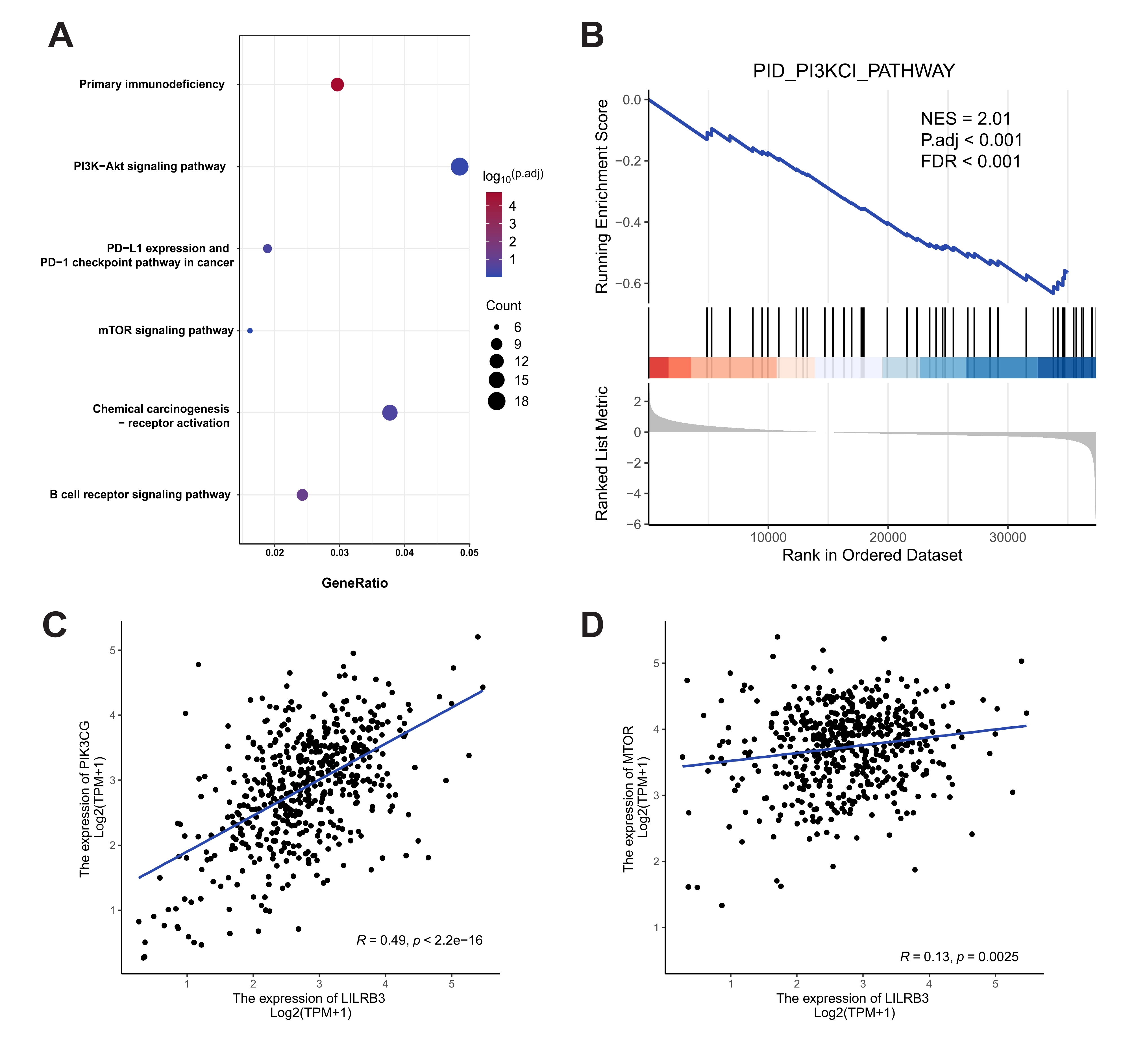

Supplement: Supplemental Material [file IANN_A_2546684_SM0491.zip › suppl_data/Supplementary Figure 5.jpg]
